# Supplementary material for: Humoral profiles of toddlers and young children following SARS-CoV-2 mRNA vaccination
Source: Nat Commun. 2024 Jan 30;15:905. doi: 10.1038/s41467-024-45181-7 (PMC10827750; doi:10.1038/s41467-024-45181-7)
Supplement: Supplementary file 1 — Supplementary Information [file 41467_2024_45181_MOESM1_ESM.pdf]

Humoral profiles of toddlers and young children following SARS-CoV-2 mRNA vaccination

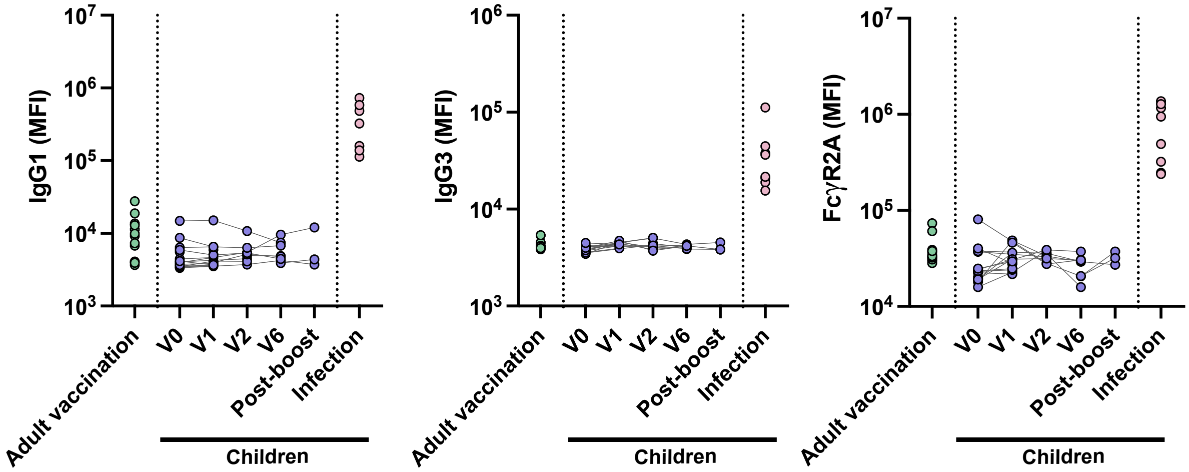

Fig. S1. Antibody response against SARS-CoV-2-specific nucleocapsid in vaccinated and infected individuals. IgG1, IgG3 and FcγR2A against nucleocapsid were measure via Luminex in adults and children that were either vaccinated with mRNA-127 or naturally infected with SARS-CoV-2. Antibody response for the infant vaccinated group is shown for V0 (before vaccination), V1 (1 month after vaccination), V2 (2 months after vaccination), V6 (6 months after vaccination) and 1 month after boosting (post-boost).

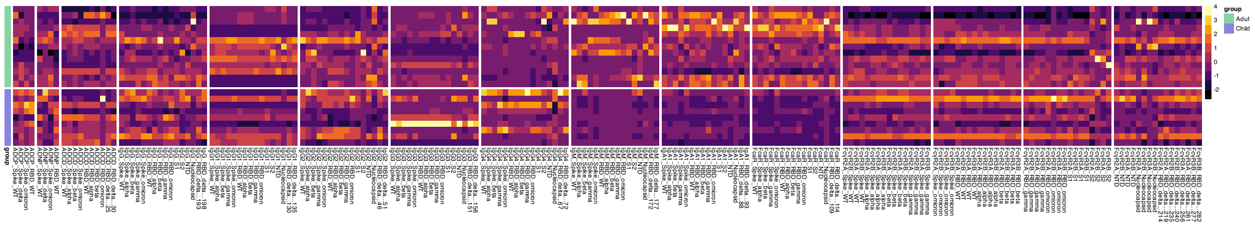

Fig. S2. mRNA-1273 vaccination induces strong humoral immunity in children less than 5 years old. Heatmap shows antibody function, antibody levels and FcR binding against SARS-CoV-2 antigens among children (n=9) and adults (n=13), 2 months after vaccination. Data were z-scored across columns and each row represent a different individual.

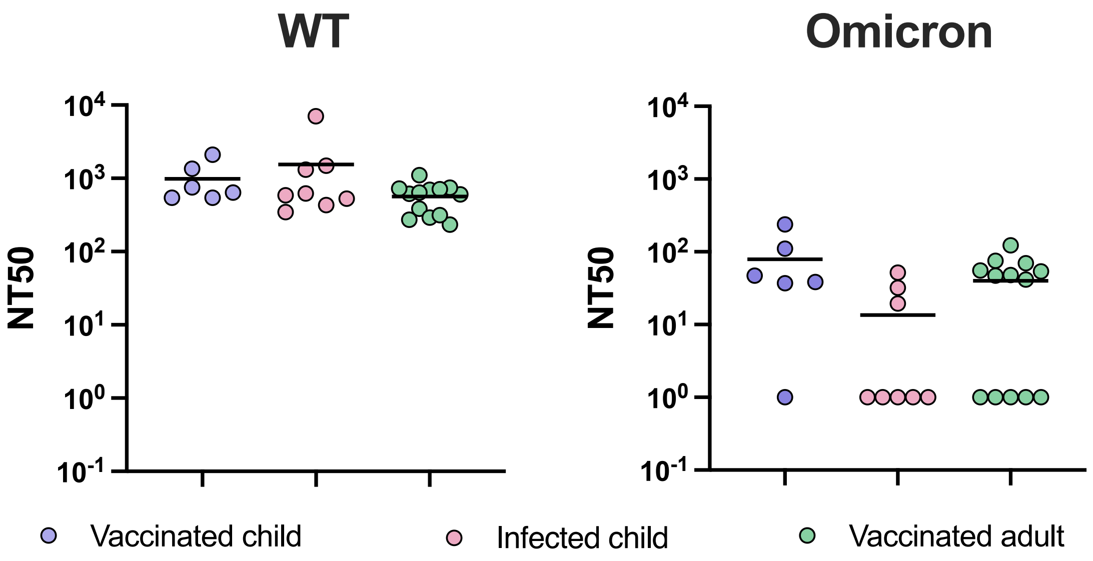

Fig. S3. Antibodoby neutralization after vaccination and infection. The dot plots show the inverse 50% pseudovirus neutralizing titers (pNT50) in vaccinated (n=6) and infected children (n=8), as well as vaccinated adults (n=13), after vaccination or infection.

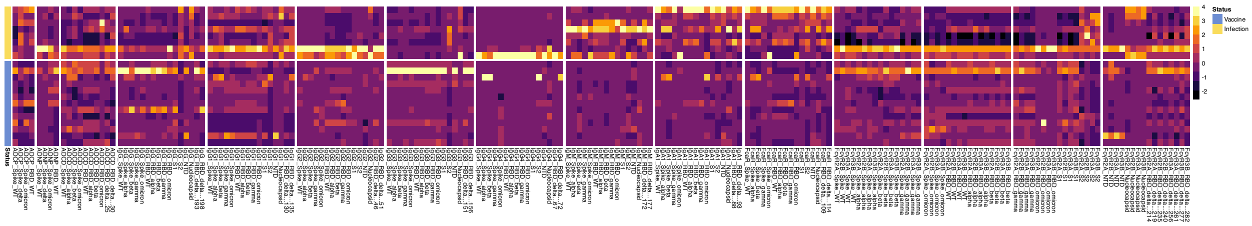

Fig. S4. Different antibody response in children less than 5 years old after SARS-CoV-2 vaccination versus infection. Heatmap shows antibody function, antibody levels and FcR binding against SARS-CoV-2 antigens among vaccinated (n=13) and infected (n=8) children. Data were z-scored across columns and each row represent a different individual.

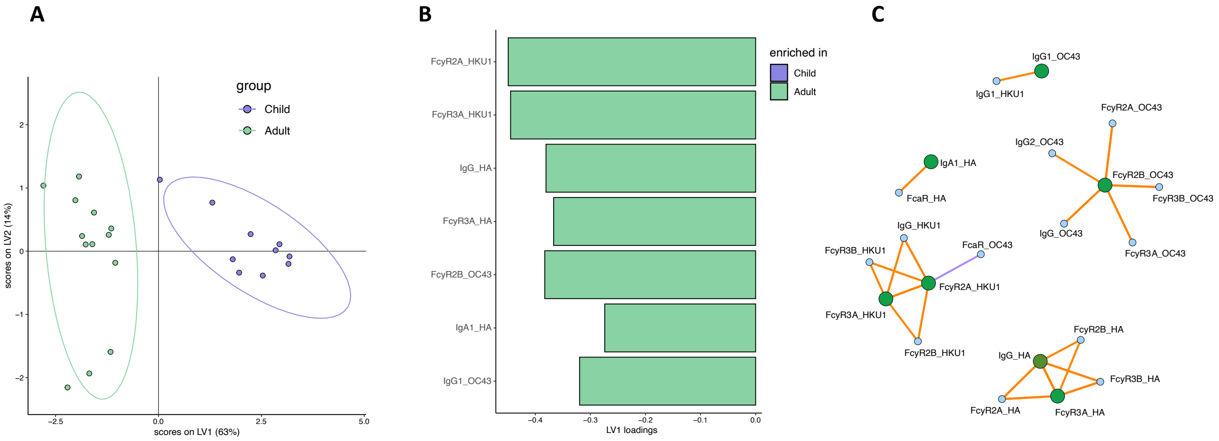

Fig. S5. Non-Sars-CoV-2 humoral immunity following mRNA-1273 vaccination in children and adults. Non-SARS-CoV-2 antibody response was measures in the plasma of children less than 5 years and and adults. PLS-DA analysis were performed (**A**) with the LASSO-selected features that were different between children and adults (**B**). Ellipses of the PLS-DA model represent 95% confidence intervals. Co-correlate analyses were performed with the LASSO-selected features and represented in a network format (**C**), with LASSO-selected features on bigger green circles, and their correlates on smaller blue circles. Correlation networks include only features with significant ($p < 0.05$) Spearman correlations, with an absolute value of correlation coefficient > 0.8. The orange lines represent positive correlation and blue lines are negative correlation. The light green dots represent the features not selected by LASSO and the dark green circles are the LASSO selected features.

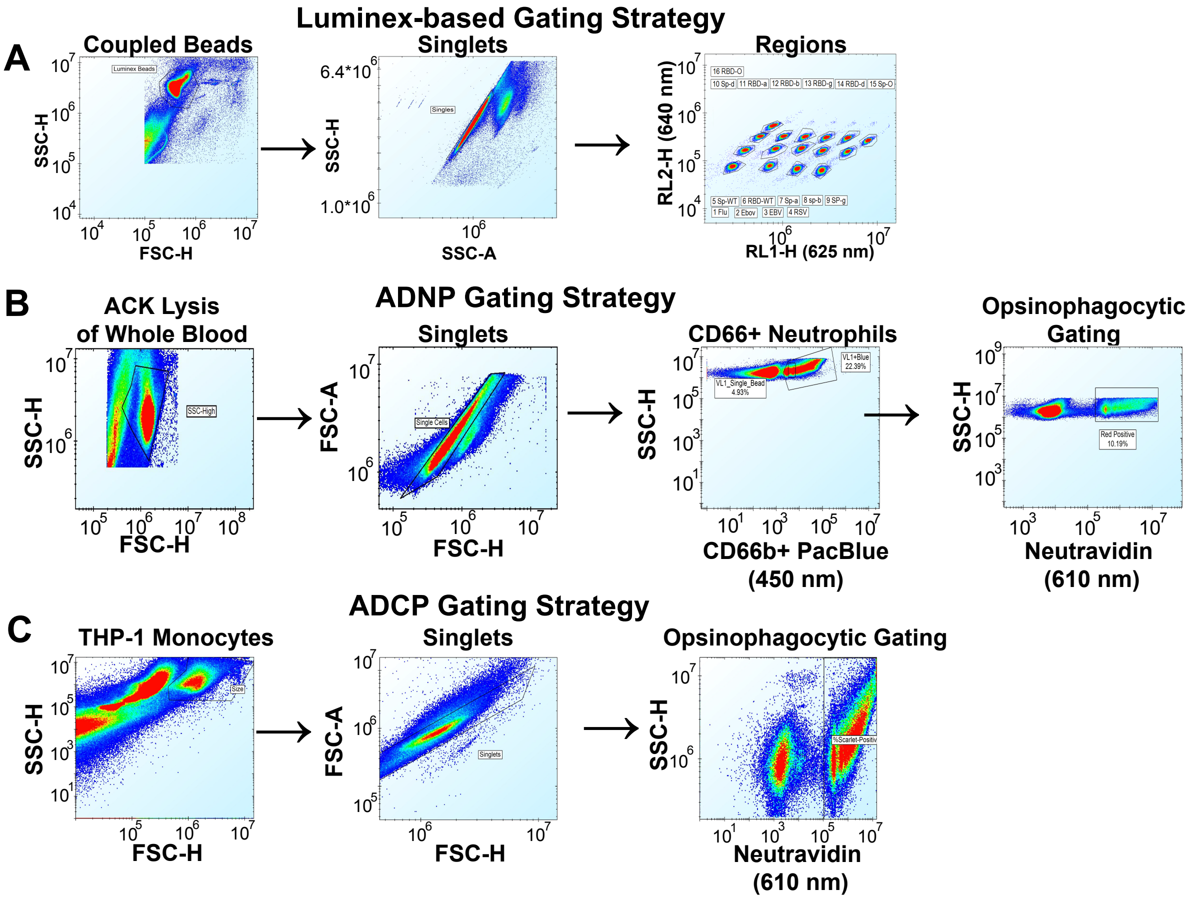

Fig. S6. Gating stategy for Luminex, ADNP, ADCP. (A) Luminex-based gating strategy to determine antibody isotype and subclass binding to a target antigen. (B) Antibody-dependent neutrophil phagocytosis (ADNP) gating strategy to determine opsinophagocytic activity towards target antigens by neutrophils that are CD66+. (C) Antibody-dependent cellular phagocytosis (ADCP) gating strategy to determine opsinophagocytic activity against target antigens by THP-1.
