# Supplementary material for: Humoral profiles of toddlers and young children following SARS-CoV-2 mRNA vaccination
Source: Nat Commun. 2024 Jan 30;15:905. doi: 10.1038/s41467-024-45181-7 (PMC10827750; doi:10.1038/s41467-024-45181-7)
Supplement: Supplementary file 3 — Reporting Summary [file 41467_2024_45181_MOESM3_ESM.pdf]

nature portfolio

Corresponding author(s): Lael M Yonker

Last updated by author(s): Nov 19, 2023

Reporting Summary

Nature Portfolio wishes to improve the reproducibility of the work that we publish. This form provides structure for consistency and transparency in reporting. For further information on Nature Portfolio policies, see our Editorial Policies and the Editorial Policy Checklist.

Statistics

For all statistical analyses, confirm that the following items are present in the figure legend, table legend, main text, or Methods section.

n/a	Confirmed	
☐	☒	The exact sample size (*n*) for each experimental group/condition, given as a discrete number and unit of measurement
☐	☒	A statement on whether measurements were taken from distinct samples or whether the same sample was measured repeatedly
☐	☒	The statistical test(s) used AND whether they are one- or two-sided<br>*Only common tests should be described solely by name; describe more complex techniques in the Methods section.*
☒	☐	A description of all covariates tested
☐	☒	A description of any assumptions or corrections, such as tests of normality and adjustment for multiple comparisons
☐	☒	A full description of the statistical parameters including central tendency (e.g. means) or other basic estimates (e.g. regression coefficient) AND variation (e.g. standard deviation) or associated estimates of uncertainty (e.g. confidence intervals)
☐	☒	For null hypothesis testing, the test statistic (e.g. *F*, *t*, *r*) with confidence intervals, effect sizes, degrees of freedom and *P* value noted<br>*Give P values as exact values whenever suitable.*
☒	☐	For Bayesian analysis, information on the choice of priors and Markov chain Monte Carlo settings
☒	☐	For hierarchical and complex designs, identification of the appropriate level for tests and full reporting of outcomes
☒	☐	Estimates of effect sizes (e.g. Cohen's *d*, Pearson's *r*), indicating how they were calculated

Our web collection on statistics for biologists contains articles on many of the points above.

Software and code

Policy information about availability of computer code

Data collection	ForeCyt® Standard Edition 8.1 was used to collect Luminex, ADNP, ADCP and ADCD assay.
Data analysis	Prism version V9.3 was used to perform statistical analysis.<br>For data analysis on R, the integrated functions in 'systemsseRology' package (https://github.com/LoosC/systemsseRology) (1.0) were used to perform feature selection and partial least-squares discriminant analysis (PLS-DA) implemented in 'ropls' package (1.22.0).

For manuscripts utilizing custom algorithms or software that are central to the research but not yet described in published literature, software must be made available to editors and reviewers. We strongly encourage code deposition in a community repository (e.g. GitHub). See the Nature Portfolio guidelines for submitting code & software for further information.

Data

Policy information about availability of data

All manuscripts must include a data availability statement. This statement should provide the following information, where applicable:

- Accession codes, unique identifiers, or web links for publicly available datasets
- A description of any restrictions on data availability
- For clinical datasets or third party data, please ensure that the statement adheres to our policy

All relevant data are included in the manuscript. Source data are provided with this paper.

Research involving human participants, their data, or biological material

Policy information about studies with human participants or human data. See also policy information about sex, gender (identity/presentation), and sexual orientation and race, ethnicity and racism.

Reporting on sex and gender	Sex at birth was collected, as documented in electronic medical record. No analyses were performed, however it was noted that 10 (37%) of the cohort was male.
Reporting on race, ethnicity, or other socially relevant groupings	Race was collected, as documented in electronic medical record. No analyses were performed, however race and ethnicity of the cohort are reported.
Population characteristics	This population was selected by age, with the pediatric participants being five years or younger.
Recruitment	Families were recruited through primary care clinics and pediatric subspecialty clinics at Mass General Hospital. Fliers were placed in clinics and on Mass General Brigham broadcast channels, and eligible families were contacted by study staff prior to clinical visits.
Ethics oversight	The protocol was approved by the MGB IRB (IRB: 2020P000955). Parental/guardian consent was obtained prior to participation.

Note that full information on the approval of the study protocol must also be provided in the manuscript.

Field-specific reporting

Please select the one below that is the best fit for your research. If you are not sure, read the appropriate sections before making your selection.

☒ Life sciences ☐ Behavioural & social sciences ☐ Ecological, evolutionary & environmental sciences

For a reference copy of the document with all sections, see nature.com/documents/nr-reporting-summary-flat.pdf

Life sciences study design

All studies must disclose on these points even when the disclosure is negative.

Sample size	We employed convenience sampling of infants and young children who were infected with SARS-CoV-2 or vaccinated with Moderna 25µg mRNA1273 vaccine at Massachusetts General Hospital (MGH). Comparable numbers of adult samples were selected based on the pediatric sample size. Based on experiences from previous studies, sample sizes are assumed to be sufficient.
Data exclusions	No data was excluded.
Replication	All experiments were run in duplicates. Results between replicates were comparable.
Randomization	Samples were randomly distributed in 96 well plates.
Blinding	Investigators were blinded during data collection and primary analysis. Group allocation was revealed once data collection and primary analysis were complete for further group-based analysis.

Reporting for specific materials, systems and methods

We require information from authors about some types of materials, experimental systems and methods used in many studies. Here, indicate whether each material, system or method listed is relevant to your study. If you are not sure if a list item applies to your research, read the appropriate section before selecting a response.

Materials & experimental systems

n/a	Involved in the study	
☐	☒	Antibodies
☐	☒	Eukaryotic cell lines
☒	☐	Palaeontology and archaeology
☒	☐	Animals and other organisms
☒	☐	Clinical data
☒	☐	Dual use research of concern
☒	☐	Plants

Methods

n/a	Involved in the study	
☒	☐	ChIP-seq
☐	☒	Flow cytometry
☒	☐	MRI-based neuroimaging

Antibodies

Antibodies used	Mouse Anti-Human IgG PE (Southern Biotech, #9040-09, clone:JDC-10)<br>Mouse Anti-Human IgG1 PE (Southern Biotech, #9052-09, clone:HP6001)<br>Mouse Anti-Human IgG2 PE (Southern Biotech, #9060-09, clone: 31-7-4)<br>Mouse Anti-Human IgG3 PE (Southern Biotech, #9210-09, clone:HP6050)<br>Mouse Anti-Human IgG4 PE (Southern Biotech, #9200-09, clone: HP6025)<br>Mouse Anti-Human IgM PE (Southern Biotech, #9020-09, clone:SA-DA4)<br>Mouse Anti-Human IgA1 PE (Southern Biotech, #9130-09, clone:B3506B4)<br>Anti-guinea pig complement C3 goat IgG fraction (MP Biomedical, #85585, polyclonal)<br>Anti-human CD66b Pacific Blue (Biolegend, # 305112, clone G10F5)
Validation	All commercial antibodies came with lot analysis, were were established and quality controlled by the manufacturer, which is as follows:<br>-Mouse anti-Human IgG PE Southern Biotech Cat#9040-09: Based on ELISA, FLISA, and flow cytometry, antibody reacts to Human/ Rhesus/Chimpanzee IgG Fc; Mr 150 kDa.<br>-Mouse anti-human IgG1 PE Southern Biotech Cat#9052-09: Based on ELISA and FLISA, antibody reacts to Human IgG1 Hinge; Mr146 kDa.<br>-Mouse anti-Human IgG2 PE Southern Biotech Cat#9060-09: Based on ELISA and FLISA, antibody reacts to Human IgG2 Fc; Mr 146 kDa.<br>-Mouse anti-human IgG3 PE Southern Biotech Cat#9210-09: Based on ELISA and FLISA, antibody reacts to the Human IgG3 Hinge; Mr 170kDa.<br>-Mouse anti-Human IgG4 PE Southern Biotech Cat#9200-09: Based on ELISA and FLISA, antibody reacts to Human IgG4 Fc; Mr 146 kDa.<br>-Mouse anti-human IgA1 PE Southern Biotech Cat#9130-09: Based on ELISA and FLISA, antibody reacts to the human IgA1 Fc; Mr170 kDa.<br>-Mouse anti-human IgM PE Southern Biotech Cat#9020-09: Based on ELISA, FLISA, and flow cytometry, antibody reacts to the human IgM; Mr900kDa.<br>- Anti-guinea pig complement C3 goat IgG fraction MP Biomedical Cat#0855371: The Biuret procedure is employed to measure total protein, utilizing bovine albumin as the standard. Antibody titer is standardized through immunoelectrophoresis with an in-house control. Specificity testing of each antiserum is conducted at a minimum of 80mg/ml via immunoelectrophoresis, revealing reactivity to guinea pig complement C3; possible cross-reactivity with other species. No antibody activity to other serum proteins is detected.<br>-Mouse anti-human CD66b Pacific Blue BioLegend Cat#305112: Based on immunofluorescent staining with flow cytometric analysis, antibody reacts to human CD66b.

Eukaryotic cell lines

Policy information about cell lines and Sex and Gender in Research

Cell line source(s)	THP-1 cells were sourced from ATCC.
Authentication	THP-1 cells were not authenticated.
Mycoplasma contamination	Cell lines were not tested for mycoplasma contamination.
Commonly misidentified lines<br>(See ICLAC register)	No commonly misidentified cell lines were used in this study.

Plants

Seed stocks	*Report on the source of all seed stocks or other plant material used. If applicable, state the seed stock centre and catalogue number. If plant specimens were collected from the field, describe the collection location, date and sampling procedures.*
Novel plant genotypes	*Describe the methods by which all novel plant genotypes were produced. This includes those generated by transgenic approaches, gene editing, chemical/radiation-based mutagenesis and hybridization. For transgenic lines, describe the transformation method, the number of independent lines analyzed and the generation upon which experiments were performed. For gene-edited lines, describe the editor used, the endogenous sequence targeted for editing, the targeting guide RNA sequence (if applicable) and how the editor was applied.*
Authentication	*Describe any authentication procedures for each seed stock used or novel genotype generated. Describe any experiments used to assess the effect of a mutation and, where applicable, how potential secondary effects (e.g. second site T-DNA insertions, mosiacism, off-target gene editing) were examined.*

Flow Cytometry

Plots

Confirm that:

☒ The axis labels state the marker and fluorochrome used (e.g. CD4-FITC).

☒ The axis scales are clearly visible. Include numbers along axes only for bottom left plot of group (a 'group' is an analysis of identical markers).

☒ All plots are contour plots with outliers or pseudocolor plots.

☒ A numerical value for number of cells or percentage (with statistics) is provided.

Methodology

Sample preparation	-ADNP: after phagocytosis incubation, primary neutrophils from healthy donors were stained with anti-CD66b. Cells were washed with PBS and fixed with 4% PFA. Cells were washed with PBS after fixation, resuspended in PBS and stored at 4C in the dark until flow cytometry analysis.<br><br>-ADCP: THP-1 were pelleted and fixed with 4% PFA, then washed with PBS after fixation. Cells were then resuspended in PBS and stored at 4C in the dark until flow cytometry analysis.
Instrument	IntelliCyt® iQue Screener PLUS.
Software	ForeCyt® Standard Edition 8.1 was used to collect Luminex, ADNP, ADCP and ADCD assay.
Cell population abundance	-Primary neutrophils were identified based on CD66b surface expression. Variations were observed based on blood donors, but usually CD66b expression was >95% within single cell gate.<br><br>-All single cells were considered THP-1 cells.
Gating strategy	A detailed description of the gating strategy is shown in Supplemental figure 6. All events were gated for granulocytes using FSC-H and SSC-H, and single cells subsequently selected using SSC-A and SSC-H.<br>For ADNP assay, primary neutrophils were selected based on CD66b expression. For ADCP, phagocytic THP-1 cells were directly identified in the single cell population.

☒ Tick this box to confirm that a figure exemplifying the gating strategy is provided in the Supplementary Information.
